# Gut Microbiota Dysbiosis and Toxic Metabolite Pathways Linked to Childhood Obesity in Eastern China

**DOI:** 10.3390/toxics13110929

**Published:** 2025-10-30

**Authors:** Ruijing Zhou, Mengyuan Zhu, Minjian Chen

**Affiliations:** 1 Gulou District Center for Disease Control and Prevention, Nanjing 210015, China; ruijingzhou28@163.com; 2 Key Laboratory of Public Health Safety and Emergency Prevention and Control Technology of Higher Education Institutions in Jiangsu Province, Department of Occupational Medicine and Environmental Health, School of Public Health, Nanjing Medical University, Nanjing 211166, China; tanshuizhu123@outlook.com; 3 State Key Laboratory of Reproductive Medicine and Offspring Health, Center for Global Health, School of Public Health, Nanjing Medical University, Nanjing 211166, China; 4 Key Laboratory of Modern Toxicology of Ministry of Education, School of Public Health, Nanjing Medical University, Nanjing 211166, China

**Keywords:** gut microbiota, childhood obesity, toxicology, tryptophan metabolism, thiamine metabolism, urban environment, 16S rRNA sequencing, toxicology network construction

## Abstract

Childhood obesity is a newly emerging public health and an emerging concern in environmental health in rapidly urbanized areas of China. This preliminary study investigated the gut microbiome composition and toxic metabolite pathways of school-aged children in Nanjing. Using 16S rRNA sequencing and PICRUSt2-based functional predictions, we observed significant microbial structural changes between the normal weight group and the overweight/obese group, although α diversity was similar. Overweight and obese children exhibited a markedly higher Firmicutes/Bacteroidetes ratio as well as an enrichment of genera such as *Subdoligranulum*, *Ruminococcus*, and *Lachnospira*, indicating increased energy harvesting and inflammation. Functionally, the downregulation of tryptophan metabolism in obese children suggests a reduction in anti-inflammatory indole and an increase in the production of pro-inflammatory kynurenine. In contrast, the upregulation of thiamine metabolism may be linked to enhanced carbohydrate utilization and lipid biosynthetic activity. Our toxicology network analysis and molecular docking experiments suggest that *AhR* and thiamine-related metabolic enzymes are targets of tryptophan and thiamine metabolism, respectively, and that *PPARG* is also a potential molecular target mediating thiamine metabolism in childhood obesity. These findings highlight the environment–microbiome–host axis as a potential pathway for metabolic toxicity in childhood obesity. Further studies are needed to validate these toxicological mechanisms and identify microbial biomarkers for early intervention.

## 1. Introduction

In recent years, rapid economic growth and nutritional transitions in China have been accompanied by a marked increase in the height and weight of children and adolescents across both urban and rural regions [1,2]. However, parallel lifestyle and dietary changes, including a shift toward high-fat, high-sugar, and processed foods, have contributed to the rising prevalence of childhood obesity. In 2019, the combined overweight and obesity rate among Chinese children and adolescents aged 6–17 years reached 19% [3]. Childhood obesity is associated not only with cardiovascular diseases, type 2 diabetes, metabolic syndrome, non-alcoholic fatty liver disease, obstructive sleep apnea, and retinopathy, but also with earlier onset of puberty [4,5]. Reflecting its chronic and pathophysiological nature, obesity has been classified as an “Adiposity-Based Chronic Disease” (ABCD) [6]. Given its multifactorial etiology, childhood obesity has become not only a pressing public health issue but also an emerging environmental health and toxicological concern globally.

The gut microbiota, a complex community of microorganisms residing in the intestine, plays a central role in host metabolism, immunity, and responses to environmental stressors. It is integral not only to nutrient absorption and the metabolism of polysaccharides, cholesterol, and choline, but also to the regulation of immune function via bacterial metabolites such as short-chain fatty acids, bile acids, and defensins [7,8,9]. Importantly, many of these microbial metabolites have toxicological significance: dysregulated short-chain fatty acids can promote metabolic toxicity, bile acid imbalance may impair intestinal and hepatic function, and alterations in tryptophan-derived metabolites (e.g., indole, kynurenine) are linked to inflammation and neurotoxicity. Growing research suggests that gut microbiota dysbiosis may mediate the adverse effects of environmental exposures—including dietary patterns, pollutants, and xenobiotics—on obesity development [10]. For example, a cross-sectional study by Bervoets et al. identified significant compositional differences in the gut microbiota between obese and lean children [11]. Similarly, a birth cohort study demonstrated that the association between gut microbiota and BMI strengthens from infancy through two years of age [12], and animal models further provided causal evidence, as fecal microbiota transplantation from obese human donors to germ-free mice induced increased weight gain and metabolic disturbances [13].

The structure and diversity of the gut microbiota are shaped by age, dietary intake, health status, and environmental exposures [14,15]. Ethnic and geographic variations also lead to noticeable differences in microbial community structure, often attributable to divergent dietary patterns and food sources [16]. Nanjing, the capital of Jiangsu Province in eastern China, represents a rapidly urbanizing region in the lower Yangtze River basin. Socioeconomic and demographic transitions have driven a shift toward Western-style dietary patterns, marked by greater intake of animal meat, sugary drinks, and processed foods [17]. At the same time, urbanization is associated with reduced physical activity and higher exposure to environmental risk factors such as food additives and air pollution [18], both of which may alter gut microbiota composition and function.

Microbiota-derived metabolites resulting from chemical intake–including short-chain fatty acids, bile acids, and tryptophan catabolites from dietary–can enter the systemic circulation and exert a wide range of physiological and toxicological effects on the host [19]. This paradigm positions the gut microbiome as a source of endogenous exposure, with metabolites that have the potential to cause damage to the organism. Based on this concept, our study investigated whether gut microbiota dysregulation in childhood obesity is a source of endogenous exposure to toxicologically active metabolites. This approach. This approach expands the traditional notion of exposure from exogenous intake to microbiome-driven internal exposure, providing a new perspective on the toxicological mechanisms underlying childhood obesity.

Network toxicology is an emerging discipline at the intersection of toxicology and computational science, systems biology, and big data analysis, which aims to build toxicity prediction models or networks to assess the potential adverse effects of chemicals to humans or the environment by integrating multi-source data (such as chemical structure, gene expression, metabolic pathways, toxicity effects, etc.). When dealing with the complex relationships between genes, proteins, or other molecules, network toxicology offers a more intuitive and systematic approach that can help us not only identify key genes associated with disease but also reveal underlying molecular pathways and mechanisms [20]. Molecular docking was further used to verify the interaction between metabolites and potential targets.

**Figure 1:**
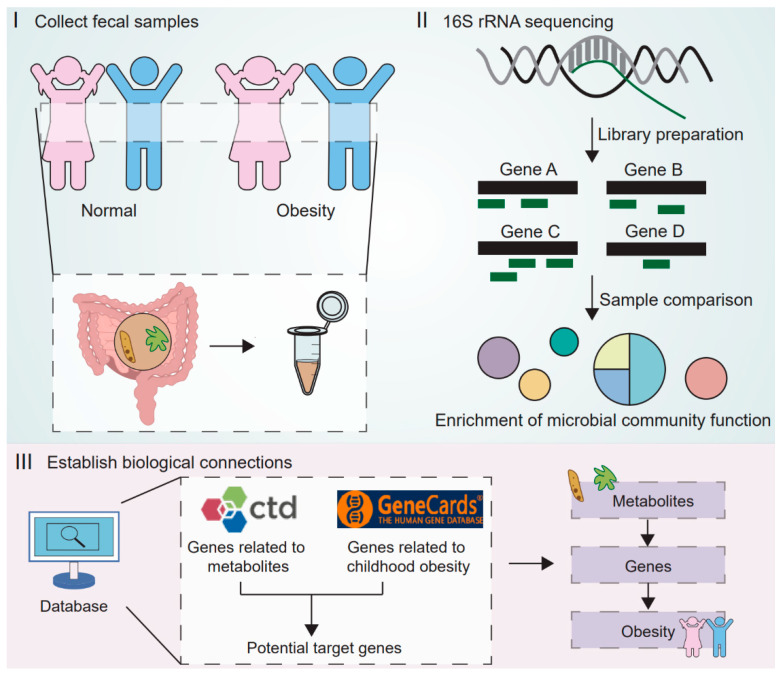
The research design diagram illustrates the three main steps of the study. The first step involves collecting fecal samples from normal weight and obese children. The second step is to perform 16S rRNA sequencing to characterize the differences in microbial composition and functions between the groups, and to predict potentially metabolism-related functional pathways. The third step integrates multiple databases (CTD and GeneCards) to establish biological connections, linking metabolites from the gut microbiome to obesity-related genes, thereby identifying potential molecular targets for childhood obesity.

To date, there has been limited research on the gut microbiota of children in eastern China, and the toxicological mechanisms by which the microbiota may influence obesity remain poorly elucidated. Therefore, this pilot study utilized 16S rRNA high-throughput sequencing to characterize the gut microbiota composition and diversity of 28 school-aged children from Gulou District, Nanjing. Our objectives were to delineate the microbial structural attributes associated with different BMI categories and to evaluate their functional implications, particularly with respect to toxicologically relevant microbial metabolites. It is anticipated that the findings from this study will provide valuable insights into the environment–microbiota–host axis and inform prevention and intervention strategies against childhood obesity and related metabolic disorders. The study design is shown in Figure 1.

## 2. Materials and Methods

### 2.1. Study Participants and Grouping

A convenience sample of third-grade students was recruited from an elementary school in Gulou District, Nanjing, China, representing children from an urban area undergoing rapid socioeconomic and nutritional transitions. Between 10 and 17 November 2020, 28 children who met the inclusion criteria were enrolled in this pilot study. In microbial–metabolic studies of childhood obesity, several preliminary omics studies have demonstrated the feasibility and value of pilot designs with sample sizes similar to or smaller than ours in generating testable mechanistic hypotheses—for example, Lindefeldt et al. (n = 12) [21,22,23].

Height and weight were measured by trained research staff using standardized protocols to calculate body mass index (BMI). Participants were classified into normal-weight, overweight, and obese groups according to the gender- and age-specific BMI cut-off values established by the Chinese National Health Standard WS/T 586-2018 (Table 1) [24]. Group differences in age and gender distribution were assessed using the one-way ANOVA and Fisher’s exact test, respectively (Table 2).

Inclusion criteria were as follows: (1) generally healthy without major diseases or gastrointestinal disorders; (2) no use of antibiotics or probiotics within three months preceding the study; (3) residency in Gulou District for at least six months, reflecting continuous exposure to the local dietary and environmental context; (4) willingness to provide fecal samples and comply with study procedures; and (5) provision of signed informed consent by a parent or guardian.

Exclusion criteria included: (1) presence of severe chronic or acute illness; (2) use of antibiotics or other medications known to influence gut microbiota composition within three months; and (3) failure to provide a fecal sample.

### 2.2. Experimental Procedures

#### 2.2.1. Questionnaire Administration and Sample Collection

Basic demographic and health information, including age, sex, health status, medication history, and dietary habits, was collected using a standardized questionnaire. These factors were considered as potential proxies for nutritional and environmental exposures. Freshly voided fecal samples (5–10 g) were collected in sterile containers and immediately stored at −20 °C within 2 h of collection until processing.

#### 2.2.2. DNA Extraction and PCR Amplification

Genomic DNA was extracted from fecal samples using the E.Z.N.A.^®^ Soil DNA Kit (Omega Bio-tek, Norcross, GA, USA) following the manufacturer’s protocol. DNA quality was verified by 1% agarose gel electrophoresis, and concentration and purity (with A260/A280 ratios between 1.8 and 2.0 considered acceptable) were determined using a NanoDrop2000 spectrophotometer (Thermo Fisher Scientific, Waltham, MA, USA). The V3–V4 region of the bacterial 16S rRNA gene was amplified with primers 338F (5′-ACTCCTACGGGAGGCAGCAG-3′) and 806R (5′-GGACTACHVGGGTWTCTAAT-3′). PCR amplification was performed on an ABI GeneAmp^®^ 9700 thermal cycler(Thermo Fisher Scientific, Waltham, MA, USA)under the following conditions: 95 °C for 3 min; 27 cycles of 95 °C for 30 s, 55 °C for 30 s, and 72 °C for 45 s; and a final extension at 72 °C for 10 min. PCR products were stored at 4 °C for further analysis.

#### 2.2.3. Illumina MiSeq Sequencing

PCR amplicons were pooled and purified using the AxyPrep DNA Gel Extraction Kit (Axygen Biosciences, Tewksbury, MA, USA) after electrophoresis on 2% agarose gels. Quantification was performed using a Quantus™ Fluorometer (Promega, Madison, WI, USA). Sequencing libraries were prepared according to Illumina’s guidelines and sequenced on a MiSeq platform with 2 × 300 bp paired-end chemistry.

#### 2.2.4. Bioinformatic Analysis

Raw sequencing reads were quality-filtered and trimmed using fastp (v0.20.0). Paired-end reads were merged using FLASH (v1.2.7). High-quality sequences were clustered into operational taxonomic units (OTUs) at a 97% similarity threshold using UPARSE (v7.1), with chimeric sequences removed. Taxonomic classification was performed using the RDP classifier (v2.2) against the SILVA 16S rRNA database (v138) with a 70% confidence threshold.

#### 2.2.5. Toxicology Network Construction

Childhood obesity-related genes were searched in the GeneCards database (https://www.genecards.org/). The Comparative Toxicogenomics Database (CTD, http://ctdbase.org/) was used to search for genes related to metabolites. We selected the overlapping genes of the two as potential research targets.

#### 2.2.6. Molecular Docking

The structure of PPARG protein (PDB:9CK0), FABP4 protein (PDB:4NNS) and AQP7 protein (PDB:8AMX) was downloaded from the PDB database. The structure of TLR4 protein (AF-000206-F1-v4) was downloaded from the AlphaFold Protein Structure Database. SDF files for thiamine diphosphate are from the PubChem database. At the same time, in order to facilitate the smooth identification of the subsequent docking software, it is necessary to convert the SDF file of the exogenous organic pollutants into the PDB file with the help of OpenBabel 2.3.2. Autodock 4.2.6 was used for molecular docking.

#### 2.2.7. Statistical Analysis

Microbial community analysis was conducted using QIIME (v1.9.1). Alpha diversity was assessed using Shannon, Simpson, Ace, and Chao1 indices. Beta diversity was evaluated through principal coordinates analysis (PCoA) based on Bray–Curtis distances. Linear discriminant analysis effect size (LEfSe) was employed to identify differentially abundant taxa (LDA score > 2.0).

After testing for normality using the Shapiro–Wilk test, group comparisons were performed as follows: Student’s *t*-test for normally distributed continuous variables in two-group comparisons, and the Kruskal–Wallis test for non-normally distributed variables or multi-group comparisons. Functional profiling was predicted using PICRUSt2 v2.2.0-b [25] with reference to the KEGG database. Particular attention was given to metabolic pathways with toxicological relevance, such as tryptophan and thiamine metabolism, due to their potential roles in inflammation and metabolic toxicity. Differential abundance analysis of predicted KEGG pathways was performed using appropriate parametric or non-parametric tests based on data distribution. All statistical tests were two-sided, with *p* < 0.05 considered statistically significant. *p*-values from multiple comparisons (including microbial taxa and functional pathways) were adjusted using the Benjamini–Hochberg false discovery rate correction. Effect sizes were reported for all significant findings to indicate the magnitude of observed differences.

## 3. Results

### 3.1. Analysis of Gut Microbiota Diversity

#### 3.1.1. Alpha Diversity

Alpha diversity was evaluated using the Shannon, Simpson, Ace, and Chao indices (Table 3). The Shannon index is positively related to microbial diversity, while the Simpson index is inversely related. The Ace and Chao indices reflect species richness and evenness. No significant differences in alpha diversity indices were observed among the three BMI groups (all *p* > 0.05, Wilcoxon rank-sum test), suggesting that the overall richness and diversity of the gut microbiota were not substantially affected. This indicates that obesity in children may be more closely linked to compositional and functional shifts, rather than overall loss of microbial diversity.

#### 3.1.2. Beta Diversity

**Figure 2:**
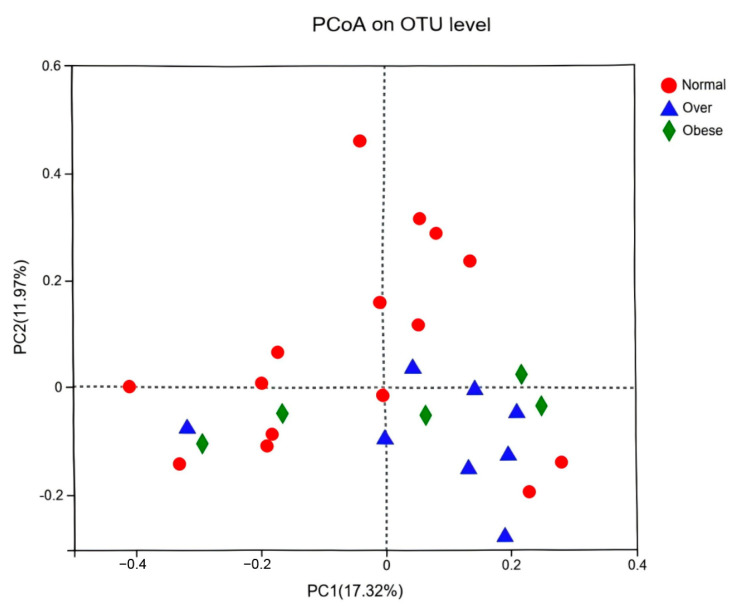
Principal coordinates analysis (PCoA) of gut microbiota at the OTU level among children with different BMI statuses. Note: Normal, normal-weight group; Over, overweight group; Obese, obese group.

Principal coordinates analysis (PCoA) based on OTU-level profiles demonstrated clear structural separation between the normal-weight group and the overweight/obese groups, while overweight and obese groups overlapped considerably (Figure 2). These findings suggest that obesity is associated with specific structural shifts in gut microbial communities, which may be influenced by common dietary and environmental exposures in the urban setting.

### 3.2. Analysis of Microbial Composition

**Figure 3:**
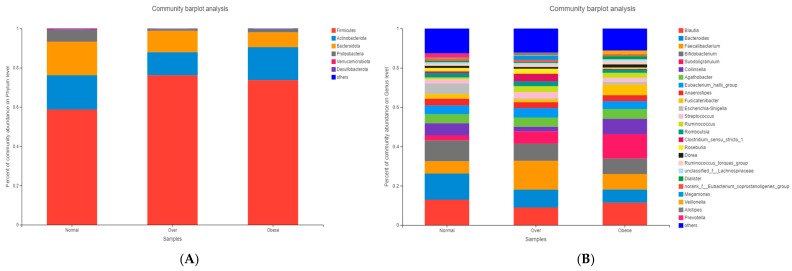
Microbial community composition of gut microbiota in children with different BMI statuses. (**A**) Composition at the phylum level; (**B**) Composition at the genus level. Note: Normal, normal-weight group; Over, overweight group; Obese, obese group.

Taxonomic composition was analyzed at the phylum and genus levels. At the phylum level (Figure 3A), *Firmicutes*, *Actinobacteriota*, and *Bacteroidota* dominated across groups. Overweight and obese children exhibited a markedly higher abundance of Firmicutes (76.36% and 73.91%, respectively) and a reduced abundance of *Bacteroidota* compared to normal-weight children (17.46%).

At the genus level (Figure 3B), the normal-weight group was enriched in *Bacteroides*, *Blautia*, and *Bifidobacterium*, taxa commonly associated with dietary fiber metabolism and gut homeostasis. In contrast, overweight and obese children exhibited higher relative abundances of *Subdoligranulum*, *Ruminococcus*, and *Lachnospira*, genera previously linked to enhanced energy harvest and inflammatory processes.

**Figure 4:**
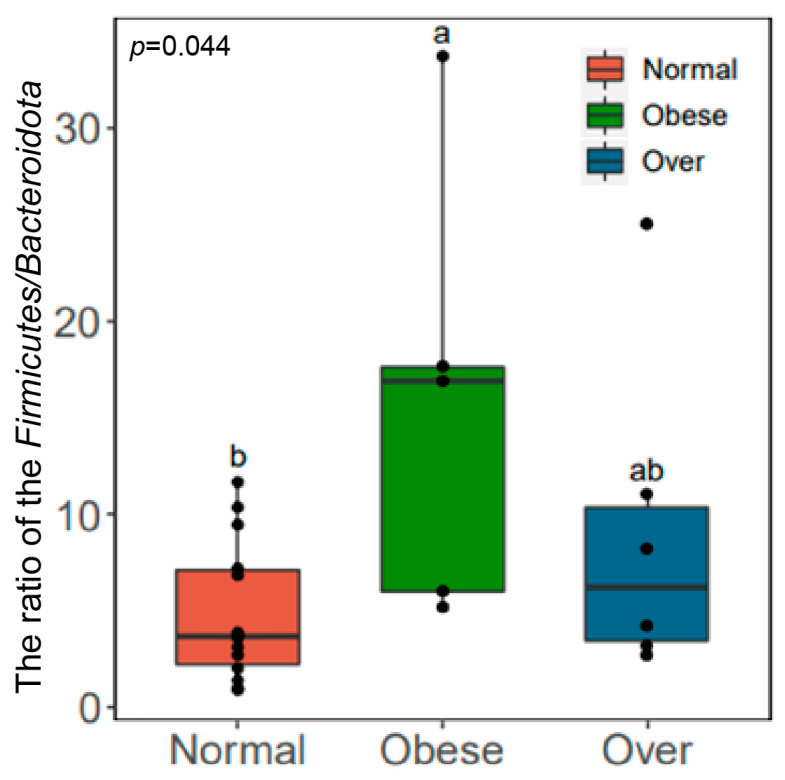
Comparison of the *Firmicutes*/*Bacteroidota* (F/B) ratio in gut microbiota among children with different BMI statuses. Note: Normal, normal-weight group; Over, overweight group; Obese, obese group; *p* < 0.05; Obese is labeled as a, Normal is labeled as b, and the difference between the two is significant; Over is labeled as ab and is not significantly different from Obese (a) or Normal (b).

The *Firmicutes*/*Bacteroidota* (F/B) ratio differed significantly among groups (*p* < 0.05, Kruskal–Wallis test). The ratio was highest in the overweight group (19.04), followed by the obese group (14.50), and lowest in the normal-weight group (10.07) (Figure 4). The observed positive association between the F/B ratio and BMI suggests that gut microbial composition could influence energy extraction efficiency, which may in turn contribute to metabolic imbalance.

### 3.3. Analysis of Intergroup Differences in Microbial Abundance

#### 3.3.1. Differential Abundance Testing

**Figure 5:**
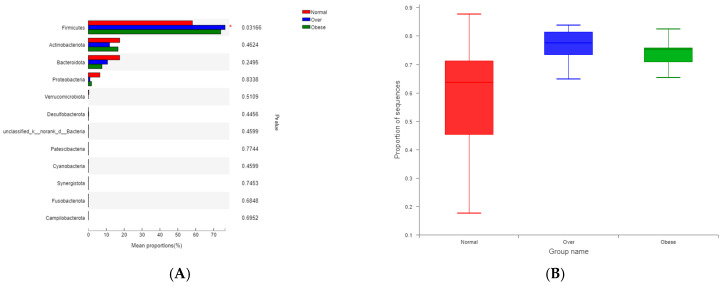

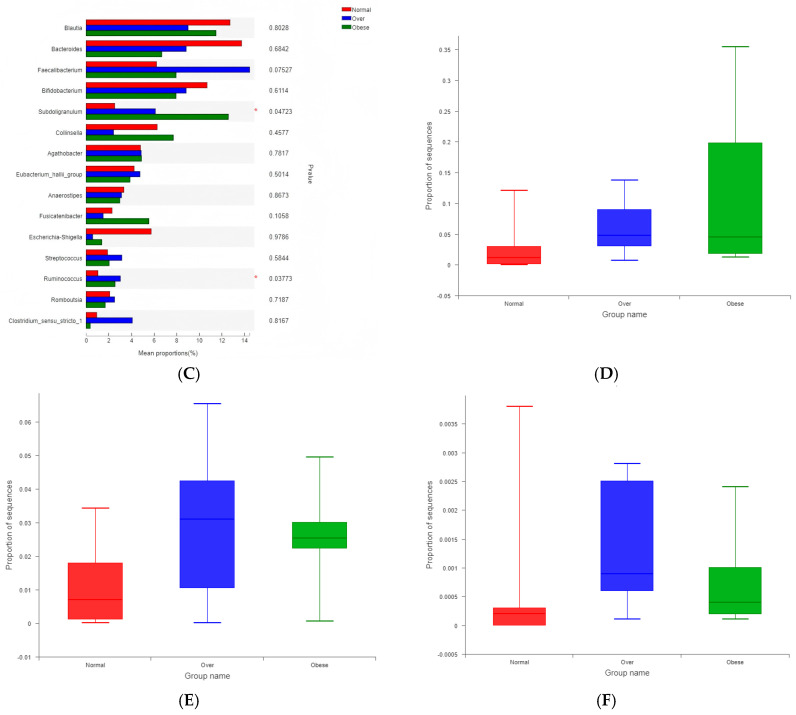
Differential abundance of gut microbial taxa among children with different BMI statuses. (**A**) Phylum-level abundance comparison; (**B**) Abundance of Firmicutes; (**C**) Genus-level abundance comparison; (**D**) Abundance of *Subdoligranulum*; (**E**) Abundance of *Ruminococcus*; (**F**) Abundance of *Lachnospira*. Note: Normal, normal-weight group; Over, overweight group; Obese, obese group. * *p* < 0.05.

At the phylum level, *Firmicutes* were significantly more abundant in both overweight and obese children compared with the normal-weight group (*p* < 0.05; Figure 5A,B). At the genus level, *Subdoligranulum*, *Ruminococcus*, and *Lachnospira* were significantly enriched in overweight and obese children (*p* < 0.05; Figure 5C–F). These genera have been implicated in metabolic imbalance and may contribute to a pro-inflammatory microenvironment.

#### 3.3.2. LEfSe Analysis

**Figure 6:**
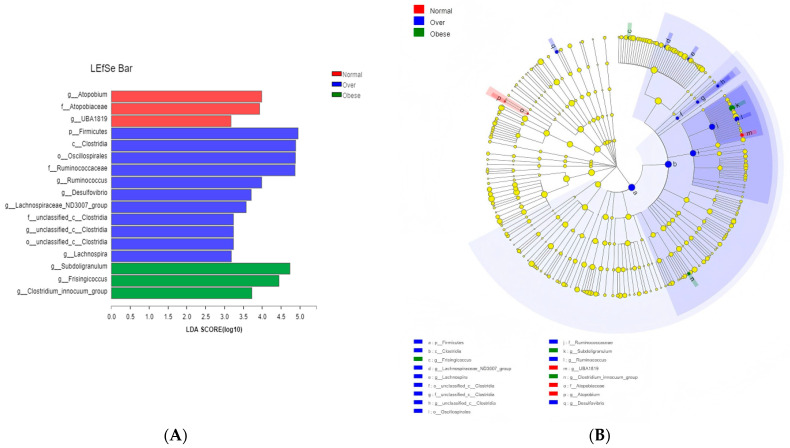
Identification of differentially abundant taxa among the three groups by LEfSe analysis. (**A**) Histogram of LDA scores; (**B**) Cladogram showing the phylogenetic distribution of discriminative features. Note: Normal, normal-weight group; Over, overweight group; Obese, obese group.

LEfSe analysis (LDA score > 2) identified enrichment of *Firmicutes*, *Clostridia*, *Oscillospirales*, and *Ruminococcaceae* in the overweight group relative to normal-weight children. The obese group exhibited enrichment of *Subdoligranulum*, *Frisingicoccus*, and the *Clostridium_innocuum group* (Figure 6A,B). These taxonomic shifts highlight potential microbial signatures of obesity with toxicological relevance, given their reported associations with host inflammation and altered metabolite production.

### 3.4. Differential Abundance of KEGG Functional Pathways

**Figure 7:**
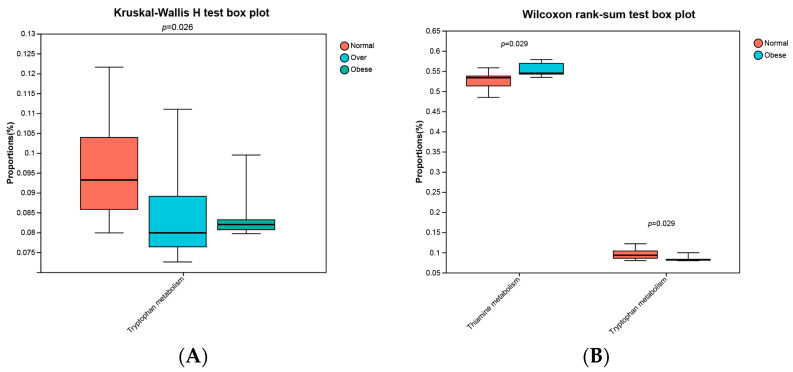
Abundances of predicted KEGG Level 3 pathways across study groups. (**A**) Kruskal-Wallis H test among three groups; (**B**) Wilcoxon rank-sum test between normal-weight and obese groups. Normal, normal-weight group; Over, overweight group; Obese, obese group. *p* < 0.05.

KEGG Level 3 pathway analysis revealed significant alterations in microbial functional predictions. Tryptophan metabolism differed among groups (*p* = 0.026, Kruskal–Wallis test), with higher levels in the normal-weight group compared to overweight and obese groups (Figure 7A). Pairwise comparisons further showed reduced tryptophan metabolism (*p* = 0.029) and increased thiamine metabolism (*p* = 0.029) in the obese group (Figure 7B).

The reduction in tryptophan metabolism suggests decreased production of protective indole, alongside potential accumulation of kynurenine-pathway metabolites with inflammatory and neurotoxic effects. Conversely, upregulated thiamine metabolism may reflect increased microbial carbohydrate catabolism and energy extraction, contributing to metabolic overload. Together, these results indicate that alterations in microbial functions with toxicological relevance may underlie the environment–microbiota–host axis in childhood obesity.

### 3.5. AhR Link Between Tryptophan Metabolites and Childhood Obesity

**Figure 8:**
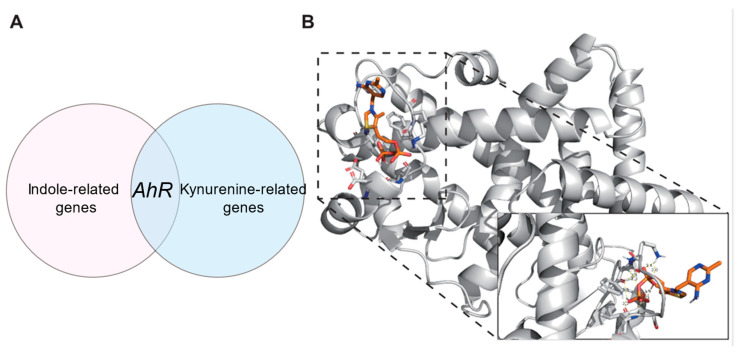
(**A**) Common target gene-*AhR*-in indole and kynurenine; (**B**) *PPARG*-TDP Molecular docking results.

Considering that the decline in tryptophan metabolic pathways leads to a decrease in indole-related metabolites and an increase in kynurenine-related metabolites, we used databases to search for related genes to explore the biological connection between metabolites and childhood obesity [26]. We searched the Comparative Toxicogenomics Database to obtain Indole and Kynurenine related genes using the keywords “Indole” and “Kynurenine”. Genes in the intersection of the CTD database were incorporated into the Indole-related gene set (Indole-set) and the Kynurenine-related gene set (Kynurenine-set). A childhood obesity-related gene set (Childhood obesity-set) was constructed with the genes retrieved from GeneCards with the keyword “Childhood obesity”. Then, the overlapping genes between the indole-set and the childhood obesity-set, as well as the overlapping genes between the kynurenine-set and the childhood obesity-set, were selected (Table 4 and Table 5). We found a common target gene–*AhR*–in indole and kynurenine (Figure 8A). In obese individuals, the level of indole is reduced, leading to the disruption of its role in enhancing the intestinal barrier and improving metabolic homeostasis after binding with *AhR*, while the accumulation of kynurenine promotes inflammation, energy imbalance, and fat deposition, ultimately resulting in obesity [27,28]. This suggests that the role of *AhR* depends on ligands and environment, and that in obesity, pro-inflammatory may be dominant.

### 3.6. PPARG Link Between Thiamine Metabolism and Childhood Obesity

As mentioned above, we searched the Comparative Toxicogenomics Database to obtain thiamine-related genes (Thiamine-set). The overlapping genes between the thiamine -set and the childhood obesity-set were selected (Table 6). Thiamine serves as coenzymes that specifically bind to thiamine-related metabolic enzymes (such as *TKT*, *NQO1*, *CAT*, *SOD1*, *CYP2E1*, etc.). Therefore, upregulation of thiamine metabolism can promote fat synthesis, leading to obesity.

Through the analysis of thiamine intersecting genes, we found that although there are many thiamine-associated metabolic enzymes (such as *TKT*, *NQO1*, *CAT*, *SOD1*, *CYP2E1*, etc.), we still identified potential target proteins, *PPARG*, *FABP4*, *AQP7*, and *TLR4*, that have small molecule binding pockets and are closely related to obesity. Therefore, we hypothesize that they can bind to TDP (Active form of thiamine), thus leading to childhood obesity. To validate this hypothesis, we used Autodock 4.2.6 software to conduct protein-ligand binding experiments. The order from low to high based on free energy is as follows: *PPARG*-Thiamine diphosphate (TDP) (−6.22 Kcal/mol) (Figure 8B), *FABP4*-*TDP* (−4.22 Kcal/mol), *AQP7*-TDP (−3.25 Kcal/mol) and *TLR4*-TDP (−2.73 Kcal/mol). According to the currently widely adopted molecular docking energy threshold (commonly ≤−5.0 Kcal/mol as a critical value for potential binding significance) [29], the binding energy of *PPARG* with TDP is significantly below this threshold, indicating that their binding has strong thermodynamic driving force and high specificity. In contrast, the interactions of other proteins with TDP did not meet this reference standard. The above experimental results suggest that *PPARG* may be a potential biological target through which TDP promotes fat accumulation and subsequently leads to childhood obesity.

## 4. Discussion

The gut microbiota plays a vital role in maintaining intestinal homeostasis and is often referred to as the “second genome” of the human body [30]. It is involved in food digestion, nutrient absorption, energy metabolism, and immune responses, and is increasingly recognized as a mediator of toxicological processes. In recent years, numerous studies have reported associations between BMI and gut microbiota composition in both adults and children. LeChatelier et al. [31] found that obese individuals had significantly lower gut microbial richness than normal-weight subjects, while Riva et al. [32] reported distinct microbial compositions in obese children. Furthermore, Lin et al. [33] demonstrated a significant correlation between BMI and beta-diversity of the gut microbiota. Our findings are consistent with recent regional investigations into childhood obesity in Eastern China. Notably, Wang et al. (2024) performed a comprehensive gut microbiota profiling of obese and normal-weight children in Southeastern China and demonstrated congruent compositional shifts, particularly an elevated Firmicutes-to-Bacteroidetes ratio [34]. While these shared structural alterations underscore a common microbial basis for obesity across the region, our study extends beyond taxonomic profiling by integrating functional prediction with toxicological network analysis and molecular docking. Thereby, our work not only corroborates regional observations but also provides novel insights into the functional and toxicological dimensions of the gut microbiome in childhood obesity.

In this study, alpha diversity indices (Shannon, Simpson, Ace, and Chao) did not differ significantly among BMI groups, indicating comparable microbial richness. However, PCoA revealed distinct beta-diversity clustering, with overweight and obese children separated from normal-weight children. These results suggest that obesity is linked to structural shifts in the gut microbiota, consistent with evidence from animal transplantation studies, where obese donor microbiota induced rapid weight gain and metabolic disturbances in germ-free mice [35,36,37]. This supports the concept that altered microbial structures may serve as pathways through which environmental and nutritional exposures exert toxicological effects on host metabolism.

Taxonomic analysis revealed *Firmicutes*, *Actinobacteriota*, *Bacteroidota*, and *Proteobacteria* as dominant phyla, with Firmicutes abundance and the *Firmicutes*/*Bacteroidota* (F/B) ratio elevated in overweight and obese children. These findings align with reports showing a positive correlation between the F/B ratio and obesity [38,39,40]. Higher F/B ratios are associated with elevated short-chain fatty acid (SCFA) production [41], which can enhance intestinal energy absorption and hepatic lipogenesis [42], contributing to metabolic overload. From a toxicological perspective, excessive SCFAs may act as “metabolic toxins,” promoting energy imbalance and low-grade inflammation. Contradictory findings from other studies reporting lower *Firmicutes* and higher *Bacteroidota* in obesity [43,44] highlight the complexity of microbiota–host interactions and the potential influence of ethnicity, diet, and environmental exposures.

At the genus level, *Subdoligranulum*, *Ruminococcus*, and *Lachnospira* were enriched in overweight and obese children, consistent with findings in obese adults [45]. LEfSe analysis identified *Firmicutes*, *Clostridia*, *Oscillospirales*, *Ruminococcaceae*, and *Subdoligranulum* as discriminative taxa. Many of these genera are associated with enhanced fermentation capacity and pro-inflammatory effects, suggesting toxicological relevance in obesity-related pathophysiology.

Functional prediction revealed downregulation of tryptophan metabolism and upregulation of thiamine metabolism in obese children. These alterations are toxicologically meaningful. Obesity is recognized as a chronic low-grade inflammatory state [46,47,48], shifting tryptophan metabolism toward the kynurenine pathway [49]. This results in tryptophan depletion and accumulation of metabolites such as kynurenine, which has inflammatory and neurotoxic properties. Reduced microbial tryptophan metabolism also impairs production of protective indole [50], which are critical for maintaining intestinal barrier integrity and immune balance. Microbiota-derived indoles serve as ligands for the aryl hydrocarbon receptor (*AhR*) [51], which regulates epithelial renewal and immune homeostasis [48]. Disruption of this pathway may exacerbate toxicant-induced intestinal vulnerability. Specifically, gene network analysis identified aryl hydrocarbon receptor (*AhR*) as a common downstream target of both indole and kynurenine pathways derived from microbial tryptophan metabolism. Our findings align with previous evidence indicating that *AhR* signaling exerts context-dependent effects. Studies have shown that in obesity, elevated kynurenine leads to *AhR*–*STAT3*–*IL-6* activation, aggravating inflammation and insulin resistance [27]. Conversely, microbial indole-derived ligands produce anti-inflammatory effects via *AhR* activation [52]. These observations suggest that the net outcome of *AhR* activation is highly ligand-specific, with kynurenine-driven pro-inflammatory signaling likely prevailing in the obese state.

Conversely, upregulation of thiamine metabolism indicates increased carbohydrate catabolism. As thiamine is a key cofactor in central carbon metabolism [53,54], elevated activity suggests excessive microbial energy harvest, potentially driving host energy surplus and fat accumulation. In terms of thiamine metabolism, thiamine-related metabolic enzymes have been identified as potential mediators of metabolic overload, with *TKT* being a key modulator in the pentose phosphate pathway (PPP) that promotes NADPH production and adipogenesis. Our molecular docking experiments further demonstrated strong binding affinities between TDP and *PPARG*—genes with established roles in lipid metabolism. The disruption of these pathways by excessive microbial thiamine metabolism may potentiate adipogenic signaling in obese children.

Several limitations of this study should be noted. Although the sample size is consistent with other preliminary omics studies [21,22,23] and the findings are supported by our extensive downstream bioinformatics analyses, which validate the rationality of our results, future studies with larger sample sizes and multi-regional cohorts are needed to verify and extend the association of these findings with childhood obesity. Second, based on microbial community data, our further mechanisms are mainly based on bioinformatics analyses and computational toxicology techniques. However, we integrated multiple bioinformatics analysis methods together with computational toxicology and molecular docking results to mutually corroborate each other and compared them with existing knowledge and literature, yielding highly consistent results, thus demonstrating their scientific validity. This paradigm is supported by a large body of literature, and researchers have successfully employed similar strategies to identify high-priority pathways for future research. For example, in previous studies, comparable sample sizes and PICRUSt2-based functional predictions were employed to generate new mechanistic hypotheses about the gut microbiome, focusing on metabolites [55]. In the future, functional experiments are needed to further validate the mechanisms we proposed.

## 5. Conclusions

Our omics study indicates that childhood obesity in eastern China is associated with the environment-microbiome-host axis. Overweight/obese children exhibited higher Firmicutes/Bacteroidetes ratios and enrichment of taxa such as *Subdoligranulum*, *Ruminococcus*, and *Lachnospiraceae*, along with reduced microbial tryptophan metabolism and increased thiamine metabolism. Multiple bioinformatics analysis methods combined with toxicology network analysis and molecular docking identified the *AhR*-indole/kynurenine axis and the *PPARG*/thiamine-associated metabolic enzymes-TDP axis as plausible mechanisms linking inflammation and energy storage. Collectively, these findings propose a mechanistic framework that connects environmental influences on the gut microbiota to adipogenic and inflammatory signaling, highlight testable microbial and metabolic biomarkers for risk stratification and early detection, and warrant further exploration and validation.

## Figures and Tables

**Table 1:** BMI cut-off values for screening overweight and obesity in children aged 8.5–9 years (kg/m^2^).

Age (Years)	Boys		Girls	
	Overweight	Obesity	Overweight	Obesity
8.5–8.9	18.1	20.3	18.1	19.9
9.0–9.9	18.5	20.8	18.5	20.4

According to the Chinese National Health Standard WS/T 586-2018.

**Table 2:** Baseline characteristics of the study participants.

Group	n	Age (Years, Mean ± SD)	Gender (M/F)
Normal	15	8.63 ± 0.30	8/7
Overweight	8	8.69 ± 0.26	3/5
Obese	5	8.70 ± 0.27	3/2
*p*-value		0.858 *	0.775 **

* One-way ANOVA, F = 0.154; ** Fisher’s exact test; M/F, Male/Female.

**Table 3:** Analysis of gut microbiota alpha diversity indices among children with different BMI statuses.

Group	Shannon (Mean ± SD)	Simpson (Mean ± SD)	Ace (Mean ± SD)	Chao (Mean ± SD)
Normal	3.12 ± 0.53	0.12 ± 0.12	251.12 ± 34.86	253.32 ± 41.60
Overweight	3.47 ± 0.37	0.06 ± 0.03	258.24 ± 33.55	266.16 ± 41.19
Obese	3.34 ± 0.23	0.08 ± 0.03	247.95 ± 47.74	249.86 ± 47.86

**Table 4:** Indole-related genes associated with childhood obesity.

Gene Symbol	Gene ID	Description
*NR1I2*	8856	Nuclear Receptor Subfamily 1 Group I Member 2
*ABCB1*	5243	ATP Binding Cassette Subfamily B Member 1
*A* *hR*	196	Aryl Hydrocarbon Receptor
*CYP3A4*	1576	Cytochrome P450 Family 3 Subfamily A Member 4
*EPO*	2056	Erythropoietin
*ARNT*	405	Aryl Hydrocarbon Receptor Nuclear Translocator
*CAT*	847	Catalase
*CYP2A6*	1548	Cytochrome P450 Family 2 Subfamily A Member 6
*MC1R*	4157	Melanocortin 1 Receptor
*MC3R*	4159	Melanocortin 3 Receptor
*MC4R*	4160	Melanocortin 4 Receptor
*MC5R*	4161	Melanocortin 5 Receptor
*PTGES*	9536	Prostaglandin E Synthase

All genes listed are protein coding genes.

**Table 5:** Kynurenine-related genes associated with childhood obesity.

Gene Symbol	Gene ID	Description
*CYP1A1*	1543	Cytochrome P450 Family 1 Subfamily A Member 1
*MAPT*	4137	Microtubule Associated Protein Tau
*A* *hR*	196	Aryl Hydrocarbon Receptor
*APP*	351	Amyloid Beta Precursor Protein
*IFNG*	3458	Interferon Gamma
*CYP1B1*	1545	Cytochrome P450 Family 1 Subfamily B Member 1
*IGFBP1*	3484	Insulin Like Growth Factor Binding Protein 1
*PRL*	5617	Prolactin
*KMO*	8564	Kynurenine 3-Monooxygenase
*PRKN*	5071	Parkin RBR E3 Ubiquitin Protein Ligase
*PSEN1*	5663	Presenilin 1
*SLC2A4*	6517	Solute Carrier Family 2 Member 4
*IDO1*	3620	Indoleamine 2,3-Dioxygenase 1
*KYNU*	8942	Kynureninase

All genes listed are protein coding genes.

**Table 6:** Thiamine-related genes associated with childhood obesity.

Gene Symbol	Gene ID	Description
*MAPT*	4137	Microtubule Associated Protein Tau
*SLC19A3*	80704	Solute Carrier Family 19 Member 3
*NFE2L2*	4780	NFE2 Like BZIP Transcription Factor 2
*NQO1* *	1728	NAD(P)H Quinone Dehydrogenase 1
*HMOX1*	3162	Heme Oxygenase 1
*IL1B*	3553	Interleukin 1 Beta
*PTGS2* *	5743	Prostaglandin-Endoperoxide Synthase 2
*SLC19A2*	10560	Solute Carrier Family 19 Member 2
*AQP7* *#*	364	Aquaporin 7
*BCL2*	596	BCL2 Apoptosis Regulator
*GCLM* *	2730	Glutamate-Cysteine Ligase Modifier Subunit
*CKM*	1158	Creatine Kinase, M-Type
*FABP4* *#*	2167	Fatty Acid Binding Protein 4
*NOS2* *	4843	Nitric Oxide Synthase 2
*TKT* *	7086	Transketolase
*TNF*	7124	Tumor Necrosis Factor
*ACHE* *	43	Acetylcholinesterase (Yt Blood Group)
*AKT1*	207	AKT Serine/Threonine Kinase 1
*BAX*	581	BCL2 Associated X, Apoptosis Regulator
*BCHE* *	590	Butyrylcholinesterase
*CAT* *	847	Catalase
*CKB* *	1152	Creatine Kinase B
*CYP2E1* *	1571	Cytochrome P450 Family 2 Subfamily E Member 1
*GSR* *	2936	Glutathione-Disulfide Reductase
*IL6*	3569	Interleukin 6
*LHB*	3972	Luteinizing Hormone Subunit Beta
*PPARG* *#*	5468	Peroxisome Proliferator Activated Receptor Gamma
*SQSTM1*	8878	Sequestosome 1
*TLR4* *#*	7099	Toll Like Receptor 4
*TNNT2*	7139	Troponin T2, Cardiac Type
*CASP1*	834	Caspase 1
*CASP3*	836	Caspase 3
*GSDMD*	79792	Gasdermin D
*HAVCR1*	26762	Hepatitis A Virus Cellular Receptor 1
*KCNH2*	3757	Potassium Voltage-Gated Channel Subfamily H Member 2
*MYC*	4609	MYC Proto-Oncogene, BHLH Transcription Factor
*NLRP3*	114548	NLR Family Pyrin Domain Containing 3
*PPARGC1A*	10891	PPARG Coactivator 1 Alpha
*RELA*	5970	RELA Proto-Oncogene, NF-KB Subunit
*SLC2A2*	6514	Solute Carrier Family 2 Member 2
*SOD1* *	6647	Superoxide Dismutase 1
*STAR*	6770	Steroidogenic Acute Regulatory Protein
*TGFB1*	7040	Transforming Growth Factor Beta 1
*TXN*	7295	Thioredoxin

*: Thiamine-associated metabolic enzymes; #: Potential target proteins; All genes listed are protein coding genes.

## Data Availability

In accordance with Articles 33–39 of Chapter 4 of the Ethical Review Measures for Biomedical Research Involving Human Subjects (2016), the consent form templates and process records have been retained for reference. Personal data cannot be publicly shared in order to protect the privacy of research participants.
